# Cumulative Fraction of Response for Once- and Twice-Daily Delamanid in Patients with Pulmonary Multidrug-Resistant Tuberculosis

**DOI:** 10.1128/AAC.01207-20

**Published:** 2020-12-16

**Authors:** Suresh Mallikaarjun, Moti L. Chapagain, Tomohiro Sasaki, Norimitsu Hariguchi, Devyani Deshpande, Shashikant Srivastava, Alexander Berg, Kuniko Hirota, Yusuke Inoue, Makoto Matsumoto, Jeffrey Hafkin, Lawrence Geiter, Xiaofeng Wang, Tawanda Gumbo, Yongge Liu

**Affiliations:** a Otsuka Pharmaceutical Development & Commercialization, Inc., Rockville, Maryland, USA; b Baylor Research Institute, Dallas, Texas, USA; c Praedicare, Dallas, Texas, USA; d Otsuka Pharmaceutical Co., Ltd., Tokushima, Japan; e University of Texas Health Science Center, Tyler, Texas, USA; f Critical Path Institute, Tucson, Arizona, USA

**Keywords:** *Mycobacterium tuberculosis*, PK-PD index, PK-PD target, cumulative fraction of response, delamanid, hollow-fiber system model of tuberculosis

## Abstract

Pharmacokinetic (PK) and pharmacodynamic (PD) analyses were conducted to determine the cumulative fraction of response (CFR) for 100 mg twice-daily (BID) and 200 mg once-daily (QD) delamanid in patients with multidrug-resistant tuberculosis (MDR-TB), using a pharmacodynamic target (PDT) that achieves 80% of maximum efficacy. First, in the mouse model of chronic TB, the PK/PD index for delamanid efficacy was determined to be area under the drug concentration-time curve over 24 h divided by MIC (AUC_0–24_/MIC), with a PDT of 252. Second, in the hollow-fiber system model of tuberculosis, plasma-equivalent PDTs were identified as an AUC_0–24_/MIC of 195 in log-phase bacteria and 201 in pH 5.8 cultures. Third, delamanid plasma AUC_0–24_/MIC and sputum bacterial decline data from two early bactericidal activity trials identified a clinical PDT of AUC_0–24_/MIC of 171. Finally, the CFRs for the currently approved 100-mg BID dose were determined to be above 95% in two MDR-TB clinical trials. The CFR for the 200-mg QD dose, evaluated in a trial in which delamanid was administered as 100 mg BID for 8 weeks plus 200 mg QD for 18 weeks, was 89.3% based on the mouse PDT and >90% on the other PDTs. QTcF (QTc interval corrected for heart rate by Fridericia’s formula) prolongation was approximately 50% lower for the 200 mg QD dose than the 100 mg BID dose. In conclusion, while CFRs of 100 mg BID and 200 mg QD delamanid were close to or above 90% in patients with MDR-TB, more-convenient once-daily dosing of delamanid is feasible and likely to have less effect on QTcF prolongation.

## Abstract (precis)

Pharmacokinetic (PK) and pharmacodynamic (PD) analyses were conducted to determine the cumulative fraction of response (CFR) for 100 mg twice-daily (BID) and 200 mg once-daily (QD) delamanid in patients with multidrug-resistant tuberculosis (MDR-TB), using a pharmacodynamic target (PDT) that achieves 80% of maximum efficacy. First, in the mouse model of chronic TB, the PK/PD index for delamanid efficacy was determined to be area under the drug concentration-time curve over 24 h divided by MIC (AUC_0–24_/MIC), with a PDT of 252.

## TEXT

Worldwide, approximately 10 million people developed tuberculosis (TB) and 1.45 million (including those coinfected with HIV) died of the disease in 2018, making it the leading cause of mortality from a single infectious agent and one of the top 10 causes of death overall (1). While drug-susceptible TB has a high cure rate following a standard 6-month regimen of antibiotics in controlled settings, global treatment success rates are 85% in routine patient care and, thus, are still not optimal (1). Management of multidrug-resistant TB (MDR-TB) is even more challenging. Accounting for approximately 460,000 cases per year worldwide, MDR-TB requires longer therapy with agents that are less effective (current rate of treatment success, 56%) and substantially more toxic (1). Additionally, resistance to either second-line injectables or fluoroquinolones (pre-extensively drug-resistant TB [pre-XDR-TB]) or to both (XDR-TB) reduces the success rate to a mere 39% (1). Thus, new therapies for effective and safe treatment of MDR-TB and XDR-TB are urgently needed.

Delamanid (Deltyba; Otsuka Pharmaceutical Co., Ltd., Tokyo, Japan) is a bicyclic nitroimidazooxazole compound that inhibits the synthesis of mycolic acids (2), key components of the lipid-rich cell wall of Mycobacterium tuberculosis (3). In preclinical studies, delamanid exhibited the lowest MIC among approved TB drugs against both drug-susceptible and drug-resistant isolates of M. tuberculosis (4) and demonstrated dose-dependent bactericidal effects in mice (2). In humans, two randomized, placebo-controlled trials assessed the efficacy and safety of delamanid plus an optimized background regimen (OBR) in the treatment of MDR-TB. In one study (trial 204; Table S5), patients who received delamanid with OBR had significantly higher sputum culture conversion (SCC) proportions after 2 months of therapy than those receiving placebo with OBR (45.4% versus 29.6%; *P* = 0.0083) (5). In the second study (trial 213; Table S5), median time to SCC was numerically shorter in the delamanid/OBR arm (51 days) than in the placebo/OBR arm (57 days), although the difference did not reach the significance level of 0.05 (*P* = 0.0562 for the comparison) (6). As of August 2020, delamanid is approved in 15 countries, including the European Union and Japan, as well as several high-TB-burden countries, including China, India, Indonesia, Peru, the Philippines, the Russian Federation, South Africa, and Ukraine, as part of an appropriate combination regimen for treatment of pulmonary MDR-TB in adults, when an effective treatment regimen cannot otherwise be composed for reasons of resistance or tolerability (7). The World Health Organization (WHO) has also recommended that delamanid be added to the WHO-recommended longer regimen in children and adolescents (6 to 17 years of age) with multidrug- or rifampin-resistant TB who are not eligible for the shorter MDR-TB regimen, under specific conditions (8).

For the treatment of infectious diseases, including TB, antibiotics should be dosed at concentrations that balance efficacy and safety. Currently, delamanid is approved at a dose of 100 mg twice daily (BID) (7) based on data from a number of clinical trials (5, 6, 9–11). A treatment regimen consisting of once-daily delamanid plus OBR has not been examined in isolation in clinical trials, but patients in trial 213 did receive 18 weeks of 200-mg once-daily (QD) dosing after 8 weeks of prior treatment with delamanid 100 mg delamanid twice a day (BID) (Table S5) (6). The present study was designed to determine the cumulative fraction of responses (CFRs) for 100 mg delamanid BID and 200 mg delamanid QD to compare the optimality of these two dosing regimens, thereby addressing the feasibility of once-daily administration of delamanid. Given the complexity of treatment regimens for MDR-TB, reducing the frequency of dosing via a once-daily regimen could have significant benefits for patient compliance, which in turn would be expected to improve outcomes for this difficult-to-treat infection.

## RESULTS

### PK/PD index and PK/PD target for delamanid in mice.

Pharmacokinetic (PK) parameters after the single administration of delamanid at doses of 0.625, 2.5, or 10 mg/kg in uninfected mice are shown in Table S1 for both plasma and lung tissue. Delamanid has a *K*_p_ (ratio of total concentration in tissue to that in plasma) value of 1.9 to 3 depending on the dose administered. Using a nonparametric superposition method, the single-dose plasma data were then used to estimate the plasma PK parameters after multiple-dose regimens, as shown in Table 1, that were used in an efficacy study (see below). The simulated PK profiles at the 4th week of a 4-week treatment for each individual regimen are shown in Fig. S2.

**TABLE 1:** Simulated PK parameters and observed bactericidal effects of various delamanid dosing regimens in M. tuberculosis Kurono-infected mice^*a*^

Regimen	Total cumulative dose (mg/kg)	AUC (mg · h/liter)		%*T*_>MIC_	*C*_max_ (mg/liter)	Log_10_ CFU/lung	
		Weekly	0–24 h			Avg value (95% CI)	Avg reduction
10 mg/kg QD	280	97.272	13.896	100	1.184	4.138 (3.329–4.948)	2.994
2.5 mg/kg BID	140	56.752	8.107	100	0.437	4.301 (4.003–4.599)	2.831
10 mg/kg 3 times/wk	120	41.688	5.955	94.8	1.039	5.655 (3.860–5.450)	2.477
2.5 mg/kg QD	70	28.376	4.054	100	0.333	4.685 (4.162–5.208)	2.447
2.5 mg/kg 3 times/wk	30	12.161	1.737	74.6	0.301	5.126 (4.391–5.862)	2.006
10 mg/kg once/wk	40	13.896	1.985	37.3	1.012	5.199 (4.793–5.605)	1.933
0.625 mg/kg QD	17.5	9.18	1.311	100	0.11	5.447 (4.855–6.040)	1.685
2.5 mg/kg once/wk	10	4.054	0.579	24.7	0.297	5.460 (5.121–5.798)	1.672
No treatment	0	0	0	0	0	7.132 (5.763–8.501)	

a The total cumulative dose was for the 4-week treatment period. Weekly AUC, *C*_max_, and %*T*_>MIC_ were estimated for the last week of the 4-week treatment period. The MIC was 0.012 mg/liter. AUC_0–24_ is the average daily AUC of that week. The log_10_ CFU/lung reduction was the average (*n* = 5, except for the regimen of 2.5 mg/kg three times per week, which had 4 mice due to the loss of one mouse resulting from a technical error) of the difference between the log_10_ CFU value for each treatment group and the average (*n* = 5) for untreated mice at the end of the 4-week treatment period. For the three-times-per-week regimen, dosing was conducted on Mondays, Wednesdays, and Fridays. For the once-a-week dose regimen, dosing was conducted on Mondays. %*T*_>MIC_, percentage of time over the treatment period when delamanid remained above the MIC (0.012 mg/liter); weekly AUC, area under the delamanid concentration-time curve over 1 week of treatment during the last week of the 4-week treatment period; *C*_max_, maximal delamanid concentration.

**FIG 1:**
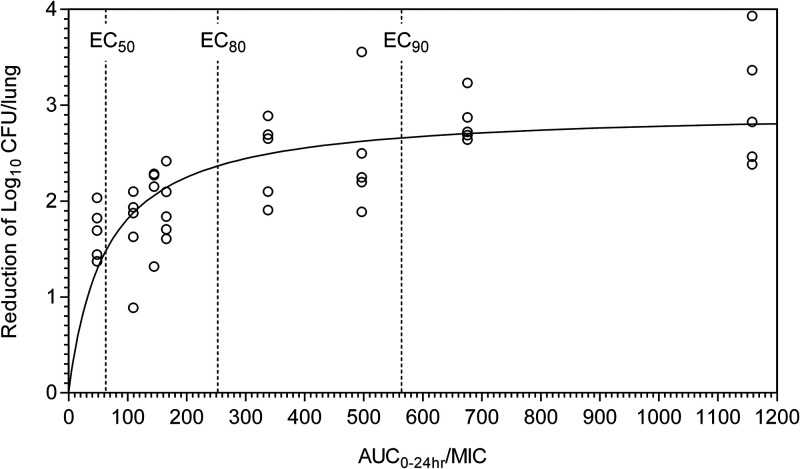
Log_10_ CFU/lung reduction by AUC_0–24_/MIC in mice. Each circle represents data from an individual mouse. The reduction was the difference between log_10_ CFU per lung in the treated mouse and the average value for the untreated mice. Multiple-dose AUC_0–24_ was simulated from single-dose PK data. EC_50_, EC_80_, and EC_90_ represent the AUC_0–24_/MIC to achieve 50%, 80%, and 90% of the maximum efficacy, respectively.

All tested delamanid dosing schedules significantly reduced bacterial burden as measured by log_10_ CFU/lung compared to untreated controls, with a minimum of 1.672 log_10_ CFU/lung reduction by the 2.5 mg/kg once-weekly treatment and a maximum of 2.994 log_10_ CFU/lung reduction by the 10-mg/kg once-daily treatment (Table 1). As shown in Table 2 and Fig. 1, the inhibitory sigmoid maximum-effect (*E*_max_) model with a fixed Hill coefficient of 1.0 fitted the data well. The *E*_max_ of delamanid in mice was estimated as a 2.96 log_10_ CFU/lung reduction (95% confidence interval [CI], 2.63 to 3.29). The ratio of area under the concentration-time curve from 0 to 24 h to MIC (AUC_0–24_/MIC) was the pharmacokinetic-pharmacodynamic (PK/PD) parameter that best described delamanid efficacy (Pearson’s correlation coefficient = 0.97; *P* < 0.0001), while the percentage of time above the MIC (%*T*_>MIC_) exhibited moderate correlation (Spearman correlation coefficient = 0.53; *P* < 0.01). There was no significant correlation between efficacy and *C*_max_/MIC by Pearson’s correlation (*P* = 0.13). Additionally, inhibitory sigmoid *E*_max_ modeling of log_10_ CFU/lung reduction versus PK/PD exposure revealed corrected Akaike information criterion scores of 2.55 (*r*^2^ = 0.99), 21.03 (*r*^2^ = 0.86), and 22.07 (*r*^2^ = 0.84) for AUC_0–24_/MIC, %*T*_>MIC_, and *C*_max_/MIC, respectively. This confirmed that AUC_0–24_/MIC was the parameter that best explained the delamanid antimicrobial effect. Therefore, the key PK/PD index driving delamanid efficacy was determined to be AUC_0–24_/MIC.

**TABLE 2:** Inhibitory sigmoid *E*_max_ parameters for delamanid AUC_0–24_/MIC from mouse and HFS studies and human EBA trials^*a*^

Study type	Estimate (95% CI) of best-fit value of:			
	*E*_max_ (log_10_ CFU)	Hill coefficient	EC_50_ (AUC_0–24_/MIC)	EC_80_ (AUC_0–24_/MIC)
Mouse	2.96 (2.63–3.28)	1 (fixed) (NE)	63 (40–99)	252 (139–649)
HFS				
Log-phase growth	1.49 (1.26–1.82)	1.86 (1.16–3.16)	240 (171–369)	506 (306–777)
pH 5.8 culture	2.35 (1.90–2.79)	1.5 (imprecise)	207 (87–361)	522 (210–910)
Human EBA	0.626 (0.448–0.835)	4.39 (1.30–8.14)	125 (57.9–213)	171 (79.0–292)

a *E*_max_, maximum killing from time-matched untreated controls in the mouse and HFS studies or from baseline in the EBA trials; EC_50_ and EC_80_, AUC_0–24_/MIC required for 50% and 80% of *E*_max_, respectively; NE, not estimated.

We then determined the pharmacodynamic target (PDT) for AUC_0–24_/MIC, using the AUC_0–24_ value that achieved 80% of the *E*_max_, a cutoff that was used in previously published studies (12–16) and validated through the translation of HFS-TB data to clinical use for various TB drugs (as detailed in the Discussion). The PDT was an AUC_0–24_/MIC of 252 (95% CI, 139 to 649) (a plasma AUC_0–24_ of 3.021 mg · h/liter divided by an MIC of 0.012 mg/liter for the M. tuberculosis strain used in the study), which achieved a 2.36 log_10_ CFU/lung reduction from the untreated control.

### PDT for delamanid from HFS-TB.

**FIG 2:**
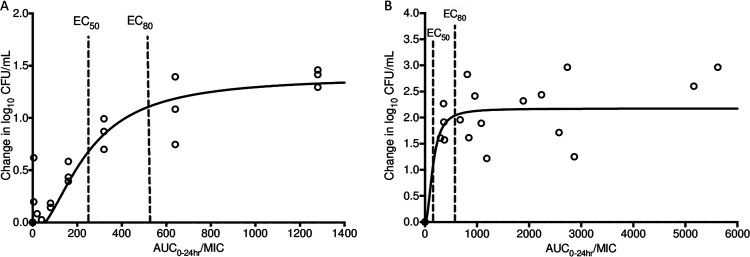
Inhibitory sigmoid *E*_max_ model for log-phase growth (A) and growth at pH 5.8 (B). An inhibitory sigmoid *E*_max_ model was used to examine the log_10_ CFU-per-milliliter values for day 7 as the response variable versus AUC_0–24_/MIC from various regimens. (A) Each circle represents data from one HFS-TB with targeted AUC_0–24_/MIC. (B) Each circle represents data from one HFS-TB with observed AUC_0–24_/MIC. EC_50_ and EC_80_ represent the AUC_0–24_/MIC to achieve 50% and 80% of the maximum efficacy, respectively.

The delamanid MIC of strain H37Rv, which was used in the study of the hollow-fiber system model for TB (HFS-TB), was determined to be 0.015 mg/liter. Maximal killing by delamanid of bacteria in log-phase growth and growth at pH 5.8 was recorded on day 7, after which all regimens failed as bacterial regrowth occurred. This regrowth in the HFS-TB has been observed for all TB drugs in monotherapy, including rifampin (17) and isoniazid (18), the two most important first-line TB drugs. The regrowth in the HFS-TB has been attributed to the emergence of resistance under monotherapy. Therefore, the killing (as calculated by the reduction of log_10_ CFU per milliliter in the delamanid treatment arms from the time-matched untreated control) at day 7 was used in the inhibitory sigmoid *E*_max_ model, as shown in Table 2 and Fig. 2. AUC_0–24_ and average log_10_ CFU-per-milliliter data for each regimen at day 7 can be found in Table S3 (for log-phase growth) and Table S4 (for growth at pH 5.8).

The EC_80_ (80% of the *E*_max_) values derived on day 7 were AUC_0–24_/MIC ratios of 506 (95% CI, 360 to 777) for log-phase growth and 522 (95% CI, 210 to 910) for growth at pH 5.8 . PDTs obtained in the HFS-TB corresponded to the drug AUCs at the site of infection (i.e., lung), while the PK/PD analyses of animal and human data were based on the plasma levels. However, there are no human data on delamanid lung penetration and the relative ratio of AUC in lung versus plasma. Hence, the *K*_p_ value could only be inferred from animal data at this time. As shown Table S1, delamanid *K*_p_ values were 1.9, 2.9, and 3.0 when delamanid was administered as a single dose at 0.625, 2.5, and 10 mg/kg, respectively. Further, it was previously shown that after a single administration of delamanid at 3 mg/kg, the *K*_p_ was 2.4 (AUC_0–24_ = 13.768 mg · h/kg in lung versus 5.673 mg · h/liter in plasma) in mice (19). Using the average of 2.6 of the 4 mouse *K*_p_ values of 1.9, 2.4, 2.9, and 3, the plasma-equivalent PDTs for log-phase growth and growth at pH 5.8 were 195 (95% CI, 139 to 299) and 201 (95% CI, 81 to 350), respectively.

### PDT for delamanid in humans.

**FIG 3:**
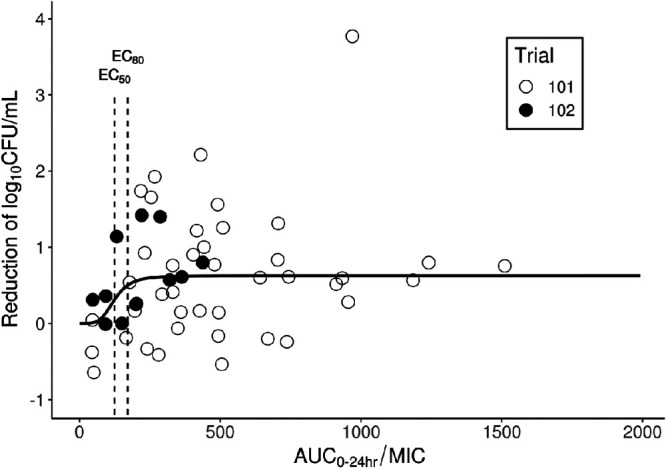
Sputum log_10_ CFU reduction from baseline (prior to the start of treatment) to the end of treatment and AUC_0–24_/MIC relationship in trial 101 and trial 102. Each circle represents data from one patient. AUC_0–24_ is the daily area under the concentration-time curve on day 14 (trial 101) or day 7 (trial 102); EC_50_ and EC_80_ represent the AUC_0–24_/MIC to achieve 50% and 80% of the maximum efficacy, respectively.

Individual delamanid AUC_0–24_/MIC and sputum culture bacterial burden reduction data from two early bactericidal activity (EBA) trials in drug-susceptible TB patients are shown in Table S6. The relationship between the reduction of log_10_ CFU per milliliter from baseline and AUC_0–24_/MIC was modeled using a nonlinear mixed-effect approach to assess the PDT of delamanid in humans. As shown in Fig. 3, an inhibitory sigmoid *E*_max_ model of bactericidal activity and plasma AUC_0–24_/MIC with random effect on maximum inhibition (*I*_max_) provided the best fit and was selected as the final model. Parameter estimates are summarized in Table 2. Goodness of fit and visual predictive check plots are provided in Fig. S4 and S5, respectively. The plasma AUC_0–24_/MIC producing 80% inhibition (i.e., the PDT) was calculated to be 171 (95% CI, 79 to 292).

### CFR in patients with MDR-TB who were treated with 100 mg delamanid BID or 200 mg delamanid QD.

As shown in Table 3, when patients were treated with 100 mg delamanid BID, the observed CFR was 100% in the trial 204 study population and ≥95% in the trial 213 study population, regardless of which PDT was used in the calculation. The CFR for 200 mg delamanid QD was 89.3% using the mouse PDT and above 90% for the other three PDTs in the trial 213 population.

**TABLE 3:** Cumulative fraction of response following 100-mg BID or 200-mg QD delamanid dosing^*a*^

Study type	Plasma-equivalent PDT (AUC_0–24_/MIC)	CFR for population and dosage		
		Trial 204, 100 mg BID (*n* = 104)	Trial 213, 100 mg BID (*n* = 246)	Trial 213, 200 mg QD (*n* = 246)
Mouse	252	100	95.9	89.3
HFS				
Log-phase growth	195^*b*^	100	98.0	93.4
pH 5.8 culture	201^*b*^	100	98.0	91.7
Human EBA	171	100	98.0	95.9

a BID, twice daily; CFR, cumulative fraction of response; EBA, early bactericidal activity; HFS-TB, hollow-fiber system model of tuberculosis; *n*, number of patients; PDT, pharmacodynamic target; QD, once daily.

b Plasma-equivalent PDT for the HFS-TB study was calculated by dividing the HFS-TB PDT with the mouse *K*_p_.

### QTcF prolongation.

In trial 213, patients with MDR-TB received 100 mg delamanid BID for 8 weeks, followed by 200 mg QD for 18 weeks (Table S5), thus providing an opportunity to compare the magnitude of corrected QT (QTc) interval prolongation under the two different dosing regimens. As shown previously, the mean differences from baseline in QTcF (QTc interval corrected for heart rate by Fridericia’s formula) between the delamanid-plus-OBR group (*n* = 341) and the placebo-plus-OBR group (*n* = 170) after 8 weeks of treatment with the 100-mg BID dose was 5.3 ms (90% CI, 2.8 to 7.9 ms) compared with 2.5 ms (90% CI, −0.3 to 5.3 ms) at the end of 18 weeks of treatment with the 200-mg QD dose (6). Thus, the QTc interval prolongation associated with 200-mg QD dosing was about 50% of that seen with 100-mg BID dosing.

## DISCUSSION

The process to develop TB drugs is highly laborious due to the complexity of TB pathology, the spatial and temporal heterogeneity of TB lesions, which drives variable microbial killing and resistance emergence, and the lack of real-time biomarkers to measure treatment outcomes (20, 21). Delamanid was identified using *in vitro* and animal TB models that demonstrated bactericidal effects on replicating, dormant, and intracellular bacilli (22). Clinical development involved the traditional drug development pathway of testing on healthy subjects first, followed by proof-of-concept and dose selection phase II trials and a phase III trial in TB patients (22). In the EBA (phase IIa) trials, 200-mg QD and 300-mg QD doses of delamanid monotherapy appeared to produce similar bactericidal activity following 14 days of treatment in patients with drug-susceptible pulmonary TB (23). Nonetheless, due to observed delamanid dose-limiting absorption (23), the phase IIb trial used BID dosing. In the trial, 100 mg BID and 200 mg BID demonstrated similar proportions of sputum culture conversion after 8 weeks of treatment when added to an OBR in pulmonary MDR-TB patients (5). Therefore, the 100-mg BID dose regimen was subsequently approved by regulatory agencies, first in 2014 by the European Medicines Agency (EMA) (22). However, insufficient data were available for a thorough PK/PD analysis of delamanid at that time, including exploring alternate dosing regimens.

In the multipronged PK/PD analyses presented here, we attempted to understand the relationship between the PK and PD of delamanid using nonclinical and human PK/PD data, based on the principles outlined in a guideline from the EMA on the use of PK/PD in the development of antimicrobial medicinal products (24). Such principles are also consistent with the FDA guidance (25) for developing antibiotics and the methodology of establishing appropriate doses for TB drugs from the WHO (26). In addition to using data from the mouse model and human EBA trials, we utilized the data from the HFS-TB model, which was recently qualified by the EMA as a method for use in support of selection and development of antituberculosis drugs (27) and is supported by the FDA as a complementary tool for dose selection (28, 29). The HFS-TB is an *in vitro* system that can mimic human PK characteristics of antimycobacterial drugs at the site of infection and was built for the purpose of exploring the concentration-effect relationships potentially relevant to the treatment of TB. Another merit of the HFS-TB is that drug efficacy can be examined under different growth conditions of M. tuberculosis bacilli (log-phase growth and low pH to simulate a slowly replicating state) and, thus, may capture some of the microbial heterogenicity of TB lesions. In addition, repetitive sampling in the HFS-TB, as in patients’ sputum, allows determination of several quantitative PD measures, including kill slopes.

AUC_0–24_/MIC is the PK/PD index for delamanid, as determined in the mouse study. In the PDT and CFR calculations, AUC_0–24_ was obtained using noncompartmental methods for data from the mouse TB model, EBA trials (trials 101 and 102), and trial 204. For the data from the HFS and trial 213, model-based approaches were applied to obtain AUC_0–24_. As shown in Fig. S3 for the HFS and the companion article (30) for trial 213, the model predictions are reasonably consistent with observations. Thus, we determined that the calculated AUCs were accurate and could be used here for PK/PD analyses. The MICs of the infecting strains used in the mouse study (MIC of 0.012 mg/liter) and the HFS-TB study (MIC of 0.015 mg/liter) are in the range of the MICs in the EBA studies (0.006 mg/liter to 0.05 mg/liter) (Table S6 and Table S7) and trial 204 (0.001 to 0.05 mg/liter) (4). The individual MICs from trial 213 have not been published, but the distribution is similar to that in trial 204.

In our PK/PD analyses, two critical cutoff values were used: EC_80_ (80% of the *E*_max_) and 90% for CFR. There is no regulatory or industry standard for a set percentage of the *E*_max_ to determine PDT. Since the *E*_max_ in the inhibitory sigmoid maximal microbial kill model is on an asymptote, a percentage of the *E*_max_ needs to be selected for the purpose of determining the PDT. Traditionally in the drug development industry, 80% or 90% of *E*_max_ has been selected (13, 31). We selected EC_80_ in our study based on 3 pieces of validation work that showed that at the level of 80%, PDTs selected from HFS-TB studies reliably predicted the drug exposures associated with clinical success. First, the accuracy to forecast optimal exposures and breakpoints in patients with TB using the EC_80_-based findings from the HFS-TB was validated by examining 20 clinical studies that were published after 26 HFS-TB experiments had been published (12). The HFS-TB EC_80_-based PDT predicted optimal drug exposures and doses that were identified in these 20 clinical studies based on agnostic machine learning analyses. The predictive accuracy of the HFS-TB in forecasting the clinical exposure values was 94.4% (95% CI, 84.3% to 99.9%). Second, a systematic analysis of HFS-TB and clinical findings indicated that EC_80_-based findings in tandem with Monte Carlo simulations for attainment of that target were similar between patients and the preclinical model (32). Third, a comprehensive analysis of all preclinical PK/PD studies in TB with clinical comparisons concluded that PDTs using 80% of *E*_max_ obtained in preclinical studies provided a reliable predictor of clinical success (31).

Importantly, increasing the efficacy level from 80% to 90% requires a large increase of AUC_0–24_, since the efficacy curve is flat from EC_80_ and above (i.e., on an asymptote). In our mouse model, the increase from EC_80_ to EC_90_ (only an additional 0.3 log_10_ CFU reduction) requires the increase of AUC_0–24_/MIC from 252 to 566 (AUC_0–24_ increases from 3.021 to 6.797 mg · h/liter) (Fig. 1), which represents very little gain in terms of microbial killing for a 2.2-fold jump in needed AUC. Doubling the AUC_0–24_ in humans from the 100-mg BID delamanid dose will likely increase side effects, such as the QTc interval prolongation. Therefore, taking into consideration the balance between efficacy and safety, we selected EC_80_ in this study. As for the cutoff of CFR, the EMA guidance provides 90% as an appropriate level for PK/PD analysis (24). FDA does not define a cutoff value for CFR as far as we are aware. However, it is generally accepted that the goal for PTA (probability of target attainment) or CFR should be at least 90% (33, 34). A white paper published in 2017 assessed the relationship between the PTA for antibacterial dosing regimens and FDA approval and concluded that, among the programs evaluated, FDA approval was granted for 88% of those achieving at least a 90% PTA versus much lower approval rates for development programs using lower percentages of PTA (35). Therefore, we felt that EC_80_ and CFR at 90% were reasonable selections for our PK/PD analyses.

We showed that the plasma or plasma-equivalent PDTs (AUC_0–24_/MIC) for delamanid ranged from 171, obtained from the human EBA trials, to 252, obtained in the mouse TB model. The PDTs from the HFS-TB assessments were 195 in log-phase growth and 201 in growth at pH 5.8. The values are within a narrow range, considering the very different models used to determine PDTs. Importantly, regardless of which PDT was used, the CFRs were >95% for the 100-mg BID dose, the dose currently approved for the treatment of pulmonary MDR-TB in adults, strongly indicating that this dose is appropriate to achieve the PDT. The similarity of the efficacy of the 200-mg BID dose evaluated in trial 204 to that of the 100-mg BID dose (5) is consistent with this conclusion.

While our analysis indicates that the 100-mg BID dose of delamanid can achieve EC_80_ in more than 90% of the patients, once-daily dosing of delamanid would be more convenient for patients and could lead to enhanced adherence. Our data show that the 200-mg QD dose could produce CFRs close to or above 90%, suggesting that 200 mg QD may be a feasible option for the MDR-TB population we evaluated. Furthermore, 200 mg QD likely causes less QTc interval prolongation than the 100-mg BID regimen, as shown in trial 213. To date, QTc interval prolongation is the major safety concern for delamanid.

The CFRs achieved with 200 mg delamanid QD are slightly lower than those from the 100-mg BID dose, and thus, once-daily doses higher than 200 mg could be explored. Data on once-daily delamanid doses higher than 200 mg, such as 300 mg, have been studied in healthy subjects, as well as in a 14-day EBA trial in pulmonary TB patients (23). In the EBA study, *C*_max_ and AUC values for 300 mg QD were higher than those for 200 mg QD but still below those achieved with the 100-mg BID dose (5). Based on delamanid exposure, QTc interval prolongation from the 300 mg QD dose would be expected to be lower than that from the 100 mg BID dose, since the concentrations of both delamanid and DM-6705, a major metabolite causing QTc interval prolongation, are lower following the 300-mg QD dose than the 100-mg BID dose (7).

Our study has several limitations. First, the mouse multiple-dose PK profiles were simulated from single-dose data in healthy animals. However, PK parameters obtained using this method were similar to the actual measurements obtained with multiple doses of delamanid in M. tuberculosis Kurono-infected mice at the dose of 2.5 mg/kg and when using the one-compartment model method (see Table S2 and the other supplemental material). Thus, the nonparametric superposition method provided a reasonable estimation of the PK parameters following multiple doses of delamanid in this study. Further, delamanid PK parameters were similar between healthy subjects and patients with TB (7). Second, different phenotypes of TB bacilli (i.e., replicating, and dormant) respond differently to drug treatment, and animal models used to generate PK/PD measures of efficacy may not faithfully mimic TB disease in humans. It should be noted, however, that the HFS-TB examined low-pH conditions, which is thought to mimic certain caseous TB lesions (36–40). Third, the mouse and HFS-TB studies were designed to evaluate bactericidal activity and not sterilizing efficacy and relapse prevention of the drug. Fourth, as shown in Fig. 3, human EBA responses in trials 101 and 102 were variable. However, the model described well the central tendency values based on goodness of fit and visual predictive check (Fig. S4 and S5). Fifth, since no human lung tissue drug concentration data are available for delamanid, we used the lung tissue and plasma levels in a mouse study to calculate *K*_p_ and obtain the plasma-equivalent HFS-TB PDT. In a separate study using guinea pigs, the *K*_p_ was 18.4 (41), which would make the plasma equivalent PDT much lower. Since human TB lesions are heterogenous, TB drug penetrations are likely lesion type dependent (42). Future studies using animals and, ideally, human TB lungs are needed to investigate delamanid lesion penetration. Finally, the nonclinical PDTs were determined with monotherapy and the human PDT was determined from short-term monotherapy in EBA trials; therefore, we cannot exclude the possibility that PDTs for each drug component in a multidrug regimen when used for MDR-TB patients could be different.

In conclusion, in the present study, we identified AUC_0–24_/MIC as the index of delamanid efficacy. Using PDTs obtained from different models, we further showed that delamanid at 100 mg BID and 200 mg QD achieved cumulative fractions of response of ≥90% in two large trials of patients with pulmonary MDR-TB. Therefore, once-daily doses of delamanid, such as 200 mg or higher, may be possible options, likely with less QTc interval prolongation than with the 100-mg BID dose. Hence, QD doses of delamanid should be further evaluated in future clinical trials.

## MATERIALS AND METHODS

### General approach to the PK/PD analysis.

To determine CFR for the 100-mg BID and 200-mg QD doses of delamanid, we used general principles for PK/PD analysis as outlined in several publications, including one from the EMA (24, 26, 33). We used data from a mouse and an HFS-TB study and several human trials for this effort (Table S5). The steps taken to obtain the CFRs are outlined in Fig. S1.

### Materials.

M. tuberculosis Kurono (ATCC 35812) was used in the mouse infection model, and H37Rv (ATCC 27294) was used in the HFS-TB studies. SLC:ICR mice were obtained from Japan SLC, Inc. (Hamamatsu, Shizuoka, Japan). The hollow-fiber cartridges with polysulfone hollow fibers were purchased from FiberCell System, Inc. (New Market, MD, USA). Delamanid was supplied by Otsuka Pharmaceutical Co., Ltd. (Tokushima, Japan).

### PK analysis of single-dose delamanid in mice and estimation after multiple doses.

All animal studies were carried out in accordance with the document “Guidelines for Animal Care and Use in Otsuka Pharmaceutical Co., Ltd.” To evaluate delamanid PK, uninfected SLC:ICR mice were administered a single dose of delamanid at 0.625, 2.5, or 10 mg/kg by oral gavage. Three to six mice in each dosage group were sacrificed at 1, 2, 4, 6, 8, 12, and 24 h to obtain blood and lung tissue. Plasma and lung concentrations of delamanid were determined by high-performance liquid chromatographic-tandem mass spectrometry (HPLC-MS/MS), according to the method described by Hirao et al. (19). Plasma AUC_0–24_ values were calculated by the linear trapezoidal method using Microsoft Excel.

Based on the data from the single-dose PK study, PK parameters from multidose delamanid at 0.625, 2.5, or 10 mg/kg were then simulated by the nonparametric superposition method using Phoenix WinNonlin software, version 6.3, in various 28-day treatment regimens—twice daily, once daily, three time per week, or once per week—the same regimens that were used in the efficacy experiments, as described below (Table 1). Results of these simulations, in combination with the delamanid MIC of 0.012 mg/liter for the M. tuberculosis Kurono strain (used in the efficacy study), were used to calculate the following PK/PD indices: weekly (504 to 672 h corresponds to the fourth week of treatment) AUC_504–672_, AUC_504–672_/MIC, daily AUC (AUC_504–672_/7), daily AUC/MIC, *C*_max504–672_, *C*_max504–672_/MIC, and %*T*_>MIC_, i.e., percentage of time above MIC during the fourth week of treatment.

### PK/PD index and PDT assessments in mice.

Mice were infected with M. tuberculosis Kurono by tail vein injection at 1.2 × 10^3^ CFU/mouse. Starting from 4 weeks postinfection (average bacterial load at the time of treatment was 5.964 log_10_ CFU/lung for 5 mice), the mice were treated with delamanid by oral gavage for 28 days using eight different dosing regimens (Table 1), with five animals per treatment group, except for one group with only four mice due to a technical error (Table 1). One group of mice did not receive delamanid and served as untreated controls. At the end of the 28-day treatment period, mice were sacrificed by exsanguination through the abdominal inferior vena cava under ether anesthesia, and lung tissue was aseptically excised. Dilutions of lung homogenates were plated on 7H11 agar medium supplemented with 10% oleic acid-albumin-dextrose-catalase (OADC), and the plates were incubated at 37°C until colonies were sufficiently grown for visual counting (usually 3 weeks). The log_10_ CFU/lung reduction in each animal was calculated by subtracting the log_10_ CFU from the mean log_10_ CFU of untreated controls.

Correlations between the various PK/PD indices and log_10_ CFU/lung reduction were analyzed by Pearson’s or Spearman correlation using SAS software, releases 9.1 and 9.3 (SAS Institute Japan Ltd., Tokyo, Japan). A *P* value less than 0.05 was considered statistically significant. Additionally, corrected Akaike information criterion scores obtained from the inhibitory sigmoid *E*_max_ modeling of log_10_ CFU/lung reduction versus PK/PD indices were further used to determine the PK/PD index for delamanid.

After the PK/PD index was determined (which is AUC_0–24_/MIC for delamanid; see Results), the PDT of delamanid in mice (that is, the AUC_0–24_/MIC required for 80% of *E*_max_ [EC_80_]) was obtained using inhibitory sigmoid *E*_max_ modeling of reduction in log_10_ CFU per lung from the average log_10_ CFU per lung of untreated mice versus the AUC_0–24_/MIC ratios for each treatment group, as follows:

(1) $${\text{log}}_{10}\text{ CFU}/\text{lung}=\frac{E_{\text{max}}\times {\left(\frac{{\text{AUC}}_{0-24}}{\text{MIC}}\right)}^{\text{Hill}}}{{\text{EC}}_{50}{}^{\text{Hill}}+{\left(\frac{{\text{AUC}}_{0-24}}{\text{MIC}}\right)}^{\text{Hill}}}$$

where *E*_max_ was the maximum log_10_ CFU/lung reduction from untreated controls, AUC_0–24_ was the average daily AUC from the last week of the 4-week treatment, MIC was 0.012 mg/liter for the infecting M. tuberculosis Kurono strain, and EC_50_ was the 50% AUC_0–24_/MIC ratio for maximum log_10_ CFU decline. The AUC_0–24_/MIC values corresponding to exposure required for 50% of *E*_max_ (EC_50_), EC_80_, and EC_90_ were calculated. These analyses were performed with SAS software, release 9.3 (SAS Institute Japan, Ltd.).

### Pharmacodynamic target assessments in the HFS-TB.

The design of the HFS-TB can be found in previous publications (43, 44) and is described briefly below. The HFS-TB was adapted for delamanid by addition of 0.1% Tween 80 and 10% bovine serum albumin to Middlebrook 7H9 broth. Delamanid concentration was measured by liquid chromatography with tandem mass spectrometry using a Waters (Milford, MA, USA) Acquity UPLC connected to a Waters Xevo TQ mass spectrometer. Data were collected using MassLynx version 4.1 SCN810 software.

Delamanid was infusion based on preliminary HFS PK experiments that identified the corresponding conditions to best mimic human PK parameters, with *C*_max_ at 4 h and a terminal half-life of 30 h to match that in humans (30 to 38 h) (7). PK parameters were derived from a one-compartment model, which provided excellent estimation of actual measured concentrations, as shown in Fig. S3. The PK model-derived AUC_0–24_ at each efficacy sampling day was then obtained for the use in the inhibitory sigmoid *E*_max_ model, as described below. Bacterial killing was studied in two M. tuberculosis culture conditions: log-phase growth and culture at pH 5.8.

### (i) Log-phase-growth study.

To prepare the inoculum, M. tuberculosis H37Rv was grown to log phase in Middlebrook 7H9 broth with 10% OADC for 4 days under shaking conditions and 5% CO_2_. On day 0, 20 ml of 6 log_10_ CFU/ml culture was inoculated into each hollow-fiber system. Starting on day 1 (average bacterial load was 6.15 log_10_ CFU/ml), doses of delamanid were administered to achieve the targets described in Table S3. These dose regimens were designed to cover the EC_80_ identified in the mouse study with sufficient exposures below and above this level to allow the establishment of an exposure-response curve. Different concentrations of delamanid were prepared in a 2-ml volume and infused over 4 h using a syringe pump. Treatment was administered daily for 28 days. The peripheral compartment of each HFS-TB was sampled on study days 3, 7, 10, 14, 21, and 28 to determine the number of bacterial CFU per milliliter. Samples for PK analysis were collected from each HFS-TB central compartment starting 15 min prior to the day 28 dose (time zero), with time points at 2, 4, 12, 20, 24, 48, and 72 h after the start of the last infusion.

### (ii) M. tuberculosis cultured at pH 5.8.

Growth at pH 5.8 was carried out as described for log-phase growth with the following modifications: nonreplicating M. tuberculosis strain H37Rv was propagated under acidic conditions (pH 5.8) before being inoculated into the HFS at an inoculum of 1 × 10^5^ (5 log_10_) CFU/ml. At day 1 of treatment initiation, the bacterial load was 5.98 log_10_ CFU/ml. The target exposures for the pH 5.8 study are shown in Table S4. Two additional exposures higher than those evaluated in the log-phase study were included in the anticipation that higher exposures were needed to kill bacilli cultured at pH 5.8.

### (iii) Efficacy determination.

Quantitative cultures were performed on bacterial samples collected from each HFS-TB peripheral compartment. The samples were obtained just before administration of the next scheduled dose of delamanid. To prevent drug carryover, each 1-ml sample was washed twice, resuspended, and serially diluted 10-fold in sterile saline for quantitative cultures. Each dilution was then cultured on antibiotic-free Middlebrook 7H10 agar plates supplemented with 10% OADC. All cultures were incubated under 5% CO_2_ at 37°C for 21 days for CFU counting.

As in the mouse study, the inhibitory sigmoid *E*_max_ model (equation 1) was used to estimate the EC_50_ and EC_80_. The MIC was 0.015 mg/liter for M. tuberculosis strain H37Rv, *E*_max_ was maximum bacterial killing from time-matched untreated controls, and the Hill coefficient was estimated for this analysis. S-ADAPT and ADAPT 5 (Biomedical Simulations Resources, University of Southern California) were used for data analysis.

### PDT assessments in trials 101 and 102.

Data from two EBA trials, trial 101 and trial 102 (Table S5), were used to assess the relationship between AUC_0–24_/MIC and bacterial kill trajectories in humans. AUC_0–24_/MIC was obtained using the individual daily AUC (trial 101, AUC on day 14; trial 102, AUC on day 7 [calculated by the linear trapezoidal rule with R version 3.2.2]) divided by the MIC for the clinical isolate from the same patient at baseline determined using the proportion method. Bacterial killing was calculated by the reduction of log_10_ CFU per milliliter from the baseline to the lowest sputum log_10_ CFU per milliliter for the 7-day treatment period in trial 101 and to the day 14 log_10_ CFU per milliliter in trial 101. The inhibitory sigmoidal *E*_max_ model (as shown in equation 1) and nonlinear mixed-effect analysis were used to model the data. The analysis was performed using NONMEM software, version 7.3.0 (ICON, Dublin, Ireland). Summary statistics and raw data used for the analysis are provided in Tables S6 and S7.

### CFRs for the 100-mg BID and 200-mg QD delamanid doses.

In trial 204, delamanid plasma concentrations were determined predose and 2, 3, 4, 10, 12, 13, 14, and 24 h postdose on days 1, 14, 28, and 56 following administration of delamanid at 100 mg BID in MDR-TB patients. Daily AUCs on days 1, 14, 28, and 56 were determined by noncompartmental analysis (NCA) methods. The average steady-state AUC_0–24_ was determined by averaging the AUC_0–24_ on days 14, 28, and 56. In trial 213, patients received 100 mg delamanid BID plus OBR for 2 months, followed by 200 mg delamanid QD plus OBR for 4 months. Due to sparse sampling of the PK in this study, the steady-state AUC_0–24_ values following the 100-mg BID and 200-mg QD dosing were estimated based on a population PK model (30). Baseline MICs for M. tuberculosis isolates from each patient were determined using the proportion method (4). Patients with M. tuberculosis isolates that had baseline MICs of ≤0.016 mg/liter were included in the analyses, as this MIC has been established as the critical concentration for delamanid (45). The calculated individual AUC_0–24_/MIC values from trial 204 and trial 213 were compared with the selected PDT values (mouse, HFS-TB plasma-equivalent, and human EBA PDTs) to determine the CFR.

### QTc interval determination.

In trial 213, 12-lead electrocardiograms (ECG) were recorded at screening baseline (day −1), at day 1, and at weekly visits from week 1 to week 12 and biweekly from week 14 to 26 during the 6-month treatment, with the subject supine and at rest for at least 10 min. During treatment, three ECGs were collected 5 to 10 min apart after the morning dose of IMP (investigational medicinal product). In addition to the initial clinical interpretation for ongoing safety evaluation by the investigator, digitally acquired ECGs were received by the central reader for processing and were analyzed by a central reader. QT intervals were corrected for heart rate using Fridericia’s formula (QTcF) and averaged from the 3 ECG readings. Delamanid’s QTc prolongation effect is mainly caused by one of its metabolites, DM-6705 (7), as DM-6705 concentrations were identified as a surrogate marker for QTc prolongation. The terminal half-life of DM-6705 is about 10 days; thus, plasma concentrations of this metabolite fluctuate very little at steady state, which is reached in about 7 weeks. Hence, the timing of performing ECGs, at or beyond 7 weeks, is not important to determine the effect of delamanid on QTc.

## Supplementary Material

Supplemental file 1
