# Supplementary material for: Cumulative Fraction of Response for Once- and Twice-Daily Delamanid in Patients with Pulmonary Multidrug-Resistant Tuberculosis
Source: Antimicrob Agents Chemother. 2020 Dec 16;65(1):e01207-20. doi: 10.1128/AAC.01207-20 (PMC7927872; doi:10.1128/AAC.01207-20)
Supplement: Supplemental file 1 [file AAC.01207-20-s0001.pdf]

Supplementary Information for

Cumulative Fraction of Response for Once- and Twice-Daily Delamanid in Pulmonary Multidrug-Resistant Tuberculosis Patients

1 Overall Study Design

Figure S1. Description of Overall PK/PD Analysis Plan.

1. Obtain PK/PD Index in a Mouse TB Model

- PK
  - Single dose PK (0.625, 2.5 and 10 mg/kg) in un-infected mice
  - Simulated multiple dose PK under various dose fractionation regimens
  - Validated the simulation by measuring the 2.5 mg/kg for 14 days in *M. tuberculosis* infected mice
- PD
  - log_{10}CFU/lung reduction of various regimens from un-treated control in *M. tuberculosis* infected mice
  - Correlation and Akaike Information Criteria score to determine the PK/PD index best explains the log_{10}CFU/lung reduction
- AUC_{0-24hr}/AUC waas determined as the PK/PD index

2. Obtain Pharmacodynamic Target (PDT)

- Mouse
  - Determine the EC_{80} (80% of the E_{max})
- HFS-TB
  - Log-phase growth
    - Reduction of log_{10}CFU/mL from untreated control
    - Inhibitory E_{max} model to determine the EC_{80}
  - pH 5.8 culture condition
    - Reduction of log_{10}CFU/mL from untreated control
    - Inhibitory E_{max} model to determine the EC_{80}
  - Convert HFS EC_{80}s to plasma-equavilent EC_{80}s
- Human EBA trials
  - PK
    - Trials 101 and 102 (intersive PK sampling)
  - PD
    - Redcution of sptutum log_{10}CFU/mL from baseline during the treatment
  - Inhibitory E_{max} model to determine the EC_{80}

3. Obtain Cumulative Fraction of Response (CFR) in Two MDR-TB Clinical Trials

- MDR-TB clinical trials
  - Trial 204; 100 mg BID
    - PK: internsive PK sampling
    - MIC determined on baseline isolates
    - Average of AUC_{0-24hr} at days 14, 28 and 56 was determined for each patient
    - AUC_{0-24h}/MIC for each patient was determined
    - Percetage of patients that can achieve the PDT from the mouse, HFS-TB, and human EBA trial was determined
- Trial 213; 100 mg BID for 8 weeks followed by 200 mg QD for 18 weeks
  - PK: sparse PK sampling. AUC_{0-24hr} at steady-state during the 100 mg BID and 200 mg QD dosing periods was obtained using a population PK model
  - MIC determined on baseline isolates
  - AUC_{0-24hr}/MIC for each patient was determined for the 100 mg BID and 200 mg QD doses
  - Percetage of patients that can achieve the PDT from the mouse, HFS-TB, and human EBA trial was determined

2 Mouse PK data

Mouse PK data in plasma and lung after a single dose of delamanid of 0.625 mg/kg, 2.5 mg/kg or 10 mg/kg are shown in **Table S1**.

Table S1. Mouse Plasma and Lung Concentration Profiles of Delamanid after Single Doses of 0.625, 2.5, or 10 mg/kg. Data are presented as mean ± standard deviation. Kp: ratio of lung $AUC_{0\text{-}24h}$ divided by plasma $AUC_{0\text{-}24h}$. BLOQ: below limit of quantification (0.006 mg/L for plasma and 0.1 mg/kg for lung tissue)

Dose (mg/kg)	Tissue type	Hours after dosing							C_{max}	$AUC_{0\text{-}24hr}$ (mgXh/L)	$AUC_{0\text{-}24hr}$ (mgXh/kg lung)	Kp
		1	2	4	6	8	12	24				
0.625	Plasma (mg/L)	0.051±0.017	0.070±0.010	0.081±0.019	0.100±0.013	0.063±0.015	0.049±0.021	0.015±0.002	0.100	1.188		1.9
	Lung (mg/kg)	0.141±0.087	0.211±0.03	0.222±0.046	0.273±0.031	0.177±0.022	0.140±0.047	BLOQ	0.273		2.258	
2.5	Plasma (mg/L)	0.133±0.030	0.193±0.040	0.220±0.020	0.297±0.083	0.167±0.028	0.166±0.049	0.049±0.012	0.297	3.581		
	Lung (mg/kg)	0.362±0.089	0.568±0.196	0.609±0.100	0.878±0.236	0.483±0.070	0.501±0.197	0.130±0.027	0.878		10.422	2.9
10	Plasma (mg/L)	0.348±0.140	0.490±0.161	1.012±0.401	0.975±0.018	0.554±0.080	0.466±0.152	0.184±0.033	1.012	11.547		
	Lung (mg/kg)	0.890±0.376	1.668±0.616	2.825±0.777	3.004±0.096	1.653±0.391	1.394±0.389	0.544±0.088	3.004		34.423	3.0

<u>Figure S2. Mouse multiple dose PK profile in the last week of the 4-week treatment (504 hours to 672 hours), simulated using the nonparametric superposition method from the single dose PK study of 0.625 mg/kg, 2.5 mg/kg, and 10 mg/kg.</u> a. 10 mg/kg once a day, b. 2.5 mg/kg twice a day, c. 10 mg/kg three times per week, d. 2.5 mg/kg once a day, e. 2.5 mg/kg three times per week, f. 10 mg/kg once per week, g. 0.625 mg/kg once a day, h. 2.5 mg/kg once per week.

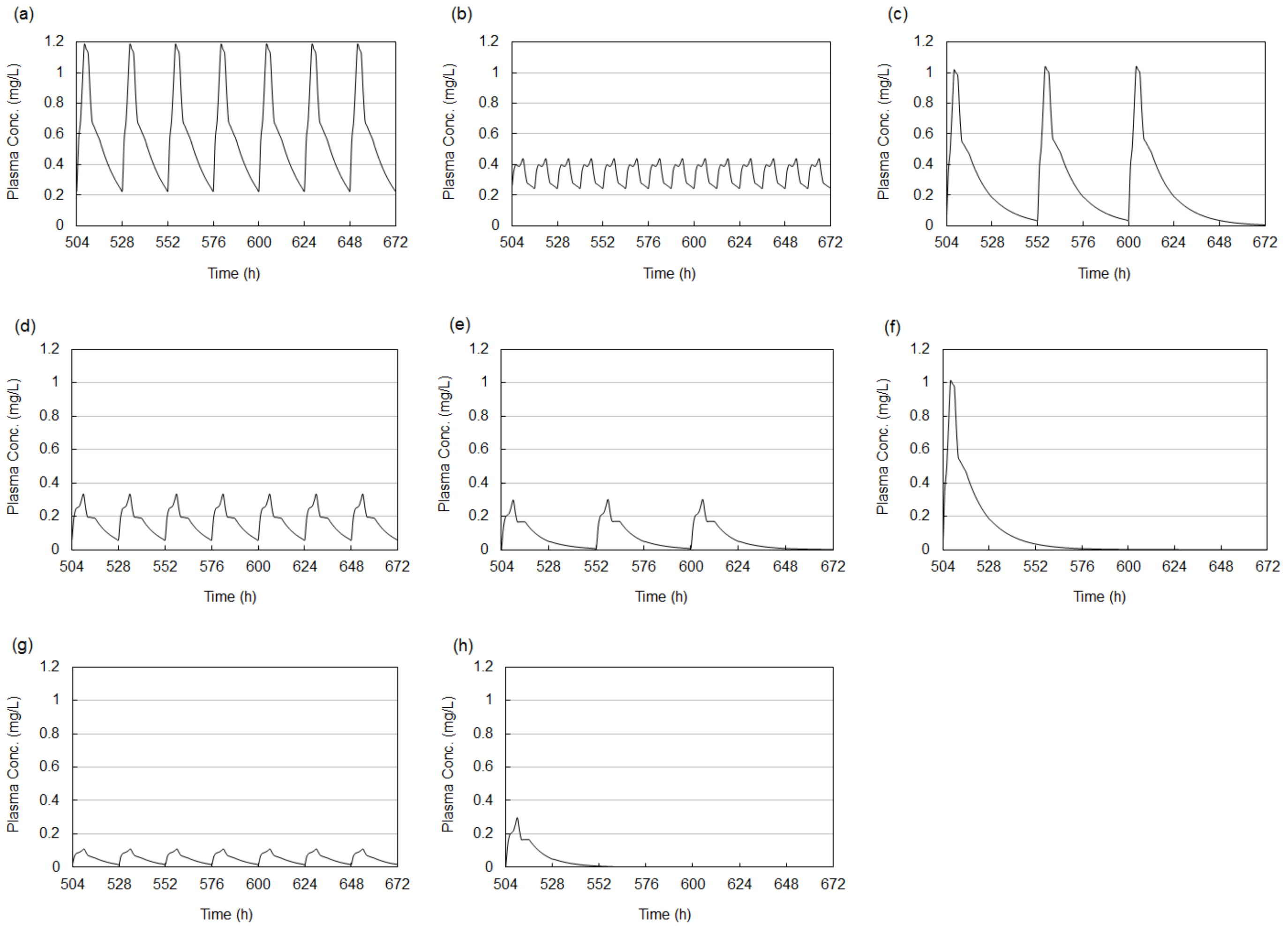

To validate the nonparametric superposition method, PK parameters following 14-day dosing of 2.5 mg/kg delamanid in *M. tuberculosis* Kurono-infected mice were determined and compared with those from the nonparametric superposition method. Blood samples were withdrawn and plasma concentrations of delamanid were determined. The plasma C_{max} and $AUC_{0\text{-}24hr}$ at the end of treatment were 0.2524 mg/L and 4.078 mg*h/L, which were similar to what was identified with the nonparametric superposition of the single dose of 2.5 mg/kg in the un-infected mice (0.333 mg/L and 4.053 mg*h/L; see **Table S1**). Further, we compared the PK parameters obtained using the nonparametric superposition method with those from the one compartment model method and identified similar parameter estimates (**Table S2**). Thus, in these studies the nonparametric superposition method provided a reasonable estimation of the PK parameters following multiple dose administration of delamanid.

Table S2. Comparison of Mouse PK Parameters Obtained using the Nonparametric Superposition Method and the One Compartmental Model Method. Model was performed for the last week of the 4-week treatment (504 hours to 672 hours). [a] same data as presented in Table 1.

Regimen	Nonparametric Superposition Model[a]		One Compartmental Model	
	$AUC_{504\text{-}672hr}$ mg×h/L	C_{max} (mg/L)	$AUC_{504\text{-}672hr}$ mg×h/L	C_{max} (mg/L)
10 mg/kg once a day	97.272	1.184	101.000	1.040
2.5 mg/kg twice a day	56.752	4.37	50.400	0.364
10 mg/kg three times per week	41.688	1.039	43.200	0.944
2.5 mg/kg once a day	28.376	0.333	25.200	0.260
2.5 mg/kg three times per week	12.161	0.301	10.800	0.236
10 mg/kg once per week	13.896	1.012	14.400	0.938
0.625 mg/kg once a day	9.180	0.110	6.300	0.065
2.5 mg/kg once per week	4.054	0.297	3.600	0.234

3 Hollow-Fiber System of Tuberculosis

3.1 HFS-TB Pharmacokinetics

A one-compartment model described well of the delamanid concentration data. The primary PK parameter, K_{el}, was a mean ± standard deviation of 0.023 ± 0.001 hr^{-1}, or a half-life of 31 ± 1.1 hours, with a calculated clearance of 0.12 ± 0.006 L/hr and volume of distribution of 5.40 ± 0.001 L. The time to maximum concentration was 4 hours. The observed versus predicted concentrations from the one-compartment model are shown in **Figure S3**. The slope was 0.998±0.002 [r^2=0.999, linear regression of observed concentrations versus model predicted concentrations], indicating no bias.

<u>Figure S3: Observed versus Predicted Delamanid Concentrations in the HFS using the One Compartment Model.</u> Each open circle is an individual time point.

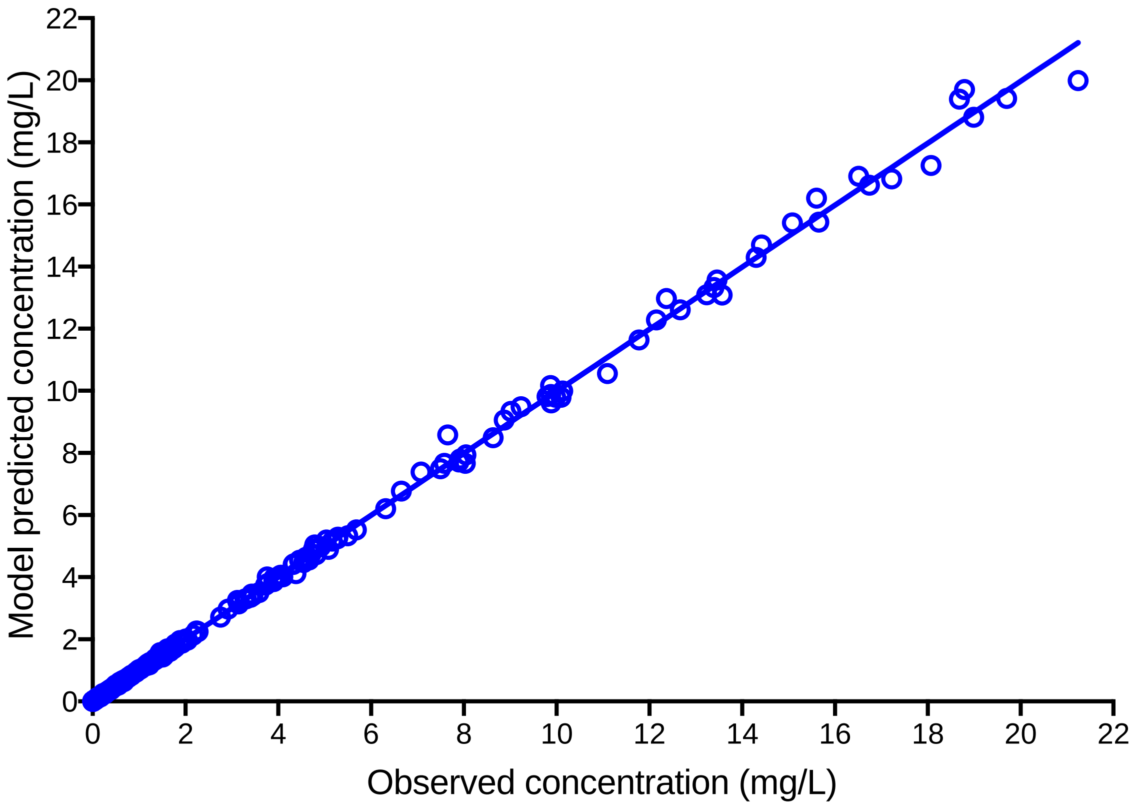

3.2 Log-Phase Growth Study

Doses of delamanid were administered to achieve the targets in the log-phase growth study, as described in **Table S3**.

Table S3. PK and PK/PD Targets to be Achieved in the Log-Phase Growth Study. $AUC_{0–24hr}$, area under the concentration-time curve from 0 to 24 hours; MIC, minimum inhibitory concentration. [a]. Peak targets estimated using a C_{max}/AUC_{0-24hr} ratio of 0.051 as seen at steady state in clinical studies. Delamanid infusion rates in the HFS-TB were adjusted to mimic these peak targets at the given AUC_{0-24hr}. [b]. Mean±standard deviation (n=3).

Regimen	Daily Dose (mg)	Peak (mg/L) [a]	$AUC_{0–24hr}$ (mg×h/L)	$AUC_{0–24hr}$/MIC	log_{10}CFU/mL at day-7[b]
1 (Control)	0	0	0	0	6.64±0.16
2	8.736	0.004	0.076	5	6.42±0.25
3	17.488	0.008	0.15	10	7.03±0.07
4	34.992	0.016	0.305	20	6.77±0.12
5	70	0.032	0.61	40	6.74±0.09
6	140	0.063	1.22	80	6.65±0.15
7	280	0.126	2.44	160	6.17±0.13
8	560	0.252	4.88	320	5.79±0.05
9	1120	0.505	9.76	640	5.57±0.22
10	2240	1.01	19.5	1280	5.26±0.08

3.3 *M. tuberculosis* Cultured at pH 5.8

The target concentrations were as shown in **Table S4**.

Table S4. Target Dosing Regimens for pH 5.8 Cultured Bacteria. $AUC_{0–24hr}$, area under the concentration-time curve from 0 to 24 hours; MIC, minimum inhibitory concentration. [a]. Peak targets estimated using a C_{max}/AUC_{0-24h} ratio of 0.051 as seen at steady state in clinical studies. Delamanid infusion rates in the HFS-TB were adjusted to mimic these peak targets at the given AUC. [b]. Mean±standard deviation (n=3).

Regimen	Daily Dose (mg)	Peak (mg/L) [a]	$AUC_{0–24hr}$ (mg×h/L)	$AUC_{0–24hr}$/MIC	log_{10}CFU/mL at day-7[b]
Non-treated control	0	0	0	0	7.89±0.20
1	140	0.063	1.22	80	5.96±0.26
2	280	0.126	2.44	160	5.92±0.23
3	560	0.252	4.88	320	6.02±0.48
4	1120	0.505	9.76	640	6.38±0.43
5	2240	1.01	19.5	1280	5.41±0.08
6	4480	2.02	39	2560	5.41±0.52
7	8960	4.04	78	5120	5.53±0.48

4 Clinical Trials

4.1 Summary of Delamanid Clinical Trials Examined in this Study

Table S5. Summary of Delamanid Clinical Trials Examined in this Study.

BID, twice daily; CFU, colony-forming unit; DLM, delamanid; MGIT, Mycobacteria growth indicator tube; NA, not applicable; OBR, optimized background regimen; QD, once daily; SCC, sputum-culture conversion. [a]. Additional activity assessments may have been performed within the individual trials. [b]. Following the 6-month treatment period, OBR was administered alone for 12 to 18 additional months.

Study	N	Intervention	Key Activity Assessments That Were Used in the PK-PD Analysis [a]	PK Sample Timing
Trial 101 (1)<br>(242-06-101) Phase II NCT00401271	11	• DLM 100 mg QD for 14 days	• PK<br>• Bactericidal activity measured by the reduction of CFU counts in sputum specimens collected overnight	Blood samples were withdrawn at time hours 0 (pre-dose), 2, 4, 6, 8, 12, and 24 hours post-dose on day 1 and 14
	10	• DLM 200 mg QD for 14 days		
	9	• DLM 300 mg QD for 14 days		
	11	• DLM 400 mg QD for 14 days		
Trial 102<br>(242-05-102) Phase II	12	• DLM 400 mg QD for 7 days	• PK<br>• Bactericidal activity measured by the reduction of CFU counts in sputum specimens collected overnight	Blood samples were withdrawn at time hours 0 (pre-dose), 2, 4, 6, 8, 12, and 24 hours post-dose on day 1 and day 7

Study	N	Intervention	Key Activity Assessments That Were Used in the PK-PD Analysis [a]	PK Sample Timing
Trial 204 (2)<br><br>(242-07-204)<br>Phase II<br>NCT00685360	141	• DLM 100 mg BID + OBR for 56 days	• PK<br>• Delamanid MIC from the baseline isolates	Blood samples were withdrawn at hours 0 (predose), 2, 3, 4, 10 (pre-evening dose),<br>12, 13, 14, and 24 on day 1, 14, 28 and 56
	136	• DLM 200 mg BID + OBR for 56 days		
Trial 213 (3) [b]<br><br>(242-09-213)<br>Phase III<br>NCT01424670	341	• DLM 100 mg BID + OBR for 2 months, followed by DLM 200 mg QD + OBR for 4 months	• Sparse PK<br>• Delamanid MIC from the baseline isolates<br>• ECG	Blood samples were withdrawn at each bi-weekly visit pre-morning dose and at a flexible time approximately 2-8 hours later point during the visit

4.2 Human EBA trials

Table S6. Raw Data Used for the Analysis from Human EBA Trials.

AUC_{0-24h}, area under the concentration-time curve from 0 to 24 hours on Day 14 (Trial 101) or Day 7 (Trial 102); MIC, minimum inhibitory concentration.

Patient #	Dose (mg)	AUC_{0-24h} (mg*hr/L)	MIC (mg/L)	AUC_{0-24h} /MIC	Baseline log_{10}CFU	log_{10}CFU reduction
**Trial 101**						
1	400	9.06	0.006	1510	6.45	0.755
2	300	4.24	0.006	706	6.92	1.31
3	400	5.76	0.012	480	7.38	0.772

Patient #	Dose (mg)	$AUC_{0–24h}$ (mg*hr/L)	MIC (mg/L)	$AUC_{0–24h}$ /MIC	Baseline log_{10}CFU	log_{10}CFU reduction
**Trial 101**						
4	200	2.78	0.012	232	6.47	0.926
5	300	5.93	0.012	494	7.01	0.143
6	100	5.47	0.006	912	7.29	0.516
7	200	6.12	0.012	510	8.02	1.26
8	100	1.53	0.006	254	6.69	1.66
9	400	3.85	0.006	642	7.03	0.601
10	300	7.44	0.006	1240	5.80	0.798
11	200	3.21	0.012	267	7.35	1.93
12	400	5.73	0.006	955	6.69	0.281
13	300	4.42	0.006	737	7.47	-0.241
14	100	2.50	0.006	416	7.62	1.22
15	200	4.22	0.006	704	5.48	0.835
16	100	2.28	0.050	45.6	7.43	0.0440
17	200	2.13	0.012	178	6.97	0.540
18	300	5.82	0.006	969	6.74	3.77
19	400	2.42	0.006	404	8.19	0.902
20	400	2.96	0.006	493	6.22	-0.165
21	100	0.610	0.012	50.8	6.25	-0.643
22	100	2.36	0.012	196	7.13	0.163
23	400	7.11	0.006	1180	7.16	0.567
24	200	4.01	0.006	669	6.18	-0.201
25	100	2.21	0.050	44.3	7.46	-0.379
26	100	2.16	0.006	360	6.83	0.149
27	300	5.60	0.006	934	6.95	0.594
28	400	5.12	0.012	427	5.92	0.165
29	200	3.97	0.012	331	6.99	0.762
30	300	4.19	0.012	349	7.01	-0.065
31	200	2.62	0.012	218	7.08	1.74
32	100	2.65	0.006	442	6.78	1.00
33	400	2.88	0.012	240	6.12	-0.334

Patient #	Dose (mg)	$AUC_{0–24h}$ (mg*hr/L)	MIC (mg/L)	$AUC_{0–24h}$ /MIC	Baseline log_{10}CFU	log_{10}CFU reduction
**Trial 101**						
34	400	4.46	0.006	743	6.93	0.612
35	300	6.07	0.012	506	6.09	-0.537
36	200	1.99	0.006	331	6.78	0.412
37	200	2.94	0.006	490	6.22	1.56
38	300	2.58	0.006	430	6.38	2.21
39	400	3.37	0.012	281	6.91	-0.412
40	100	1.99	0.012	166	6.96	-0.188
41	100	3.52	0.012	294	7.21	0.383
Trial 102						
1	400	2.18	0.006	364	6.61	0.610
2	400	3.85	0.012	321	6.97	0.570
3	400	1.72	0.006	287	7.28	1.40
4	400	2.22	0.024	92.3	6.48	-0.01
5	400	2.63	0.006	438	5.97	0.800
6	400	2.41	0.012	201	6.38	0.250
7	400	1.81	0.012	151	6.66	0
8	400	1.12	0.012	93.4	7.03	0.360
9	400	0.564	0.012	47.0	7.32	0.310
10	400	1.59	0.012	132	6.79	1.14
11	400	2.43	0.012	202	7.48	0.260
12	400	2.65	0.012	221	7.79	1.42

Table S7. Summary Statistics of Data Used for the Analysis from Human EBA trials. $AUC_{0–24hr}$, area under the concentration-time curve from 0 to 24 hours on Day 14 (Trial 101) or Day 7 (Trial 102); MIC, minimum inhibitory concentration.

**Trial**	**Dose (mg)**	**N**	**Mean**	**Min**	**Median**	**Max**
Log_{10}CFU/mL at baseline						
101	100	11	7.06	6.25	7.13	7.62
	200	10	6.75	5.48	6.88	8.02
	300	9	6.72	5.80	6.89	7.47
	400	11	6.82	5.92	6.91	8.19
102	400	12	6.90	5.97	6.88	7.79
Log_{10}CFU/mL reduction (change from baseline)						
101	100	11	0.356	-0.643	0.163	1.66
	200	10	0.976	-0.201	0.881	1.93
	300	9	0.891	-0.537	0.696	3.77
	400	11	0.340	-0.412	0.567	0.902
102	400	12	0.593	-0.0100	0.465	1.42
AUC_{0-24hr} at last dosing interval (mg*h/L)						
101	100	11	2.48	0.610	2.28	5.47
	200	10	3.40	1.99	3.07	6.12
	300	9	5.15	2.58	5.42	7.44
	400	11	4.79	2.42	4.46	9.06
102	400	12	2.10	0.564	2.20	3.85
MIC (mg/L)						
101	100	11	0.0162	0.00600	0.0120	0.0500
	200	10	0.00960	0.00600	0.0120	0.0120
	300	9	0.00800	0.00600	0.00600	0.0120
	400	11	0.00818	0.00600	0.00600	0.0120
102	400	12	0.0115	0.00600	0.0120	0.0240
AUC_{0-24hr}/MIC						
101	100	11	289	44	254	912
	200	10	393	178	331	704
	300	9	707	349	706	1241
	400	11	669	240	493	1511
102	400	12	212	47	202	438

<u>Figure S4: Goodness of Fit Plots of Human EBA Model</u>.

Blue line represents loess smoothing curve. Numbers within the plots represent ID number of each individual patient. DV: dependent variable (change in $\log_{10}$CFU), PRED: population prediction, CWRES: conditional weighted residuals.

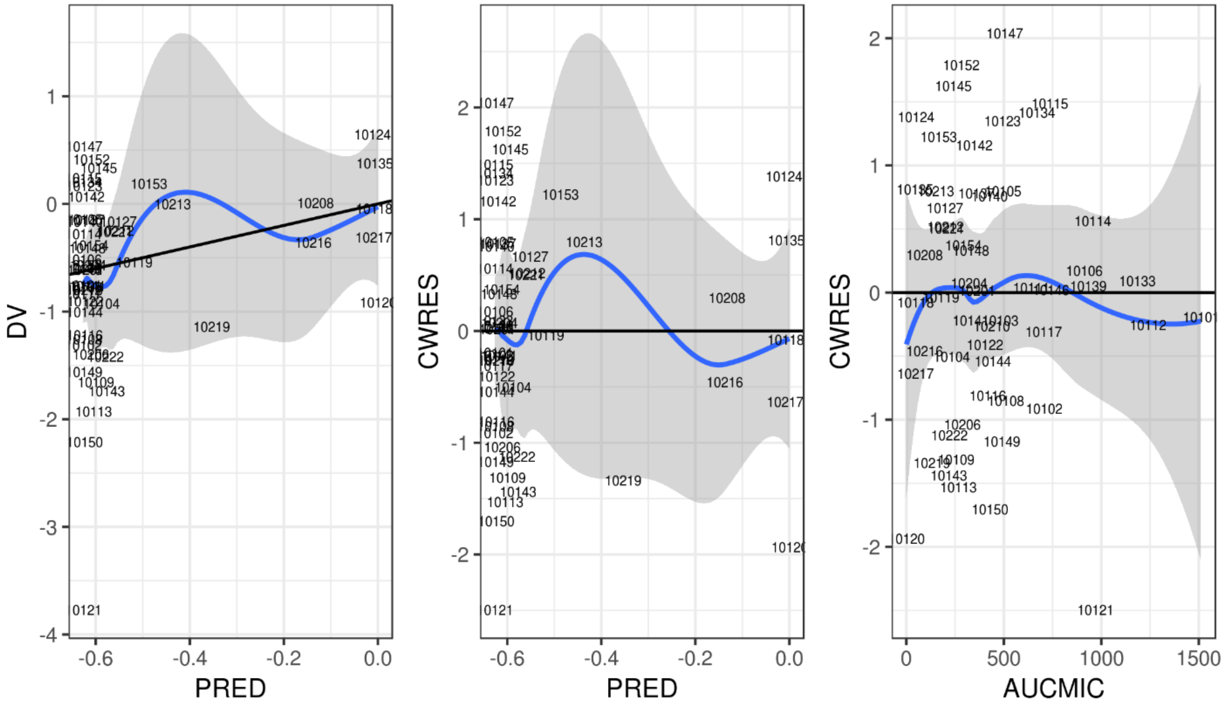

Figure S5: Visual Predictive Check Plot of Human EBA Model.

Open square represents observation. Red and black solid line represent median of observation and prediction, respectively. Red and black dashed line represent 90 percentile of observation and prediction, respectively. Shaded area represents 95% confidence interval of predictions (median [red] and 90 percentiles [blue]).

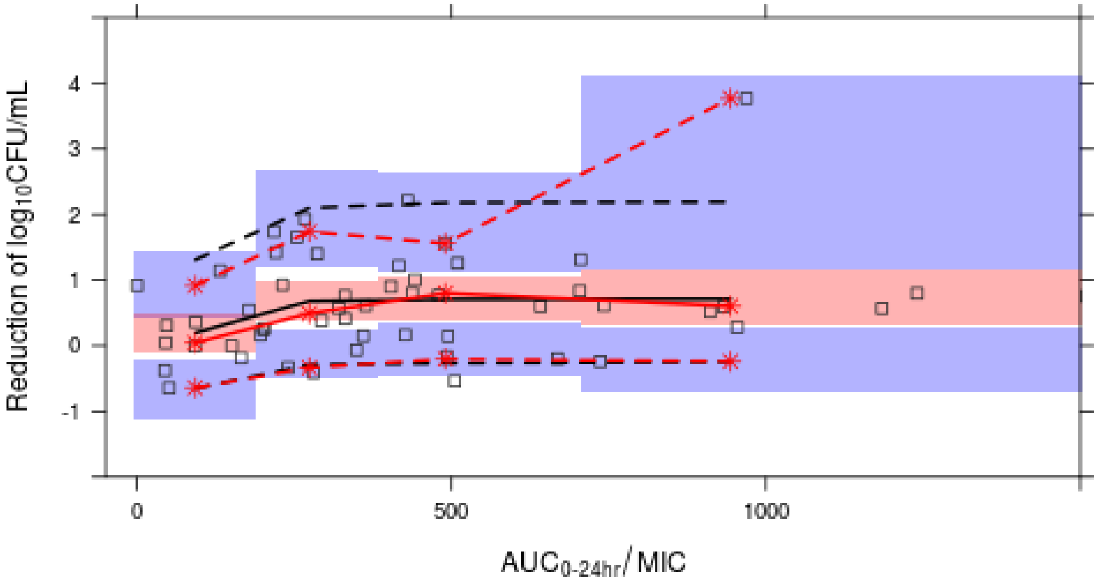

REFERENCES

1. Diacon AH, Dawson R, Hanekom M, Narunsky K, Venter A, Hittel N, Geiter LJ, Wells CD, Paccaly AJ, Donald PR. 2011. Early bactericidal activity of delamanid (OPC-67683) in smear-positive pulmonary tuberculosis patients. Int J Tuberc Lung Dis 15:949-54.

2. Gler MT, Skripconoka V, Sanchez-Garavito E, Xiao H, Cabrera-Rivero JL, Vargas-Vasquez DE, Gao M, Awad M, Park SK, Shim TS, Suh GY, Danilovits M, Ogata H, Kurve A, Chang J, Suzuki K, Tupasi T, Koh WJ, Seaworth B, Geiter LJ, Wells CD. 2012. Delamanid for multidrug-resistant pulmonary tuberculosis. N Engl J Med 366:2151-60.

3. von Groote-Bidlingmaier F, Patientia R, Sanchez E, Balanag V, Jr., Ticona E, Segura P, Cadena E, Yu C, Cirule A, Lizarbe V, Davidaviciene E, Domente L, Variava E, Caoili J, Danilovits M, Bielskiene V, Staples S, Hittel N, Petersen C, Wells C, Hafkin J, Geiter LJ, Gupta R. 2019. Efficacy and safety of delamanid in combination with an optimised background regimen for treatment of multidrug-resistant tuberculosis: a multicentre, randomised, double-blind, placebo-controlled, parallel group phase 3 trial. Lancet Respir Med 7:249-259.
